# Hexokinase 1 is required for glucose-induced repression of *bZIP63, At5g22920*, and *BT2* in *Arabidopsis*

**DOI:** 10.3389/fpls.2015.00525

**Published:** 2015-07-14

**Authors:** Sabine Kunz, Per Gardeström, Edouard Pesquet, Leszek A. Kleczkowski

**Affiliations:** Department of Plant Physiology, Umeå Plant Science Center, Umeå UniversityUmeå, Sweden

**Keywords:** glucose sensing, hexokinase, BT2 expression, bZIP63 expression, At5g22920 expression, diurnal regulation of expression, sugar regulation of gene expression

## Abstract

Simple sugars, like glucose (Glc) and sucrose (Suc), act as signals to modulate the expression of hundreds of genes in plants. Frequently, however, it remains unclear whether this regulation is induced by the sugars themselves or by their derivatives generated in the course of carbohydrate (CH) metabolism. In the present study, we tested the relevance of different CH metabolism and allocation pathways affecting expression patterns of five selected sugar-responsive genes (*bZIP63, At5g22920, BT2, MGD2*, and *TPS9*) in *Arabidopsis thaliana*. In general, the expression followed diurnal changes in the overall sugar availability. However, under steady growth conditions, this response was hardly impaired in the mutants for CH metabolizing/ transporting proteins (a*dg1, sex1, sus1-4, sus5/6*, and *tpt2*), including also hexokinase1 (HXK1) loss- and gain-of-function plants—*gin2.1* and *oe3.2*, respectively. In addition, transgenic plants carrying *pbZIP63::GUS* showed no changes in reporter-gene-expression when grown on sugar under steady-state conditions. In contrast, short-term treatments of agar-grown seedlings with 1% Glc or Suc induced *pbZIP63::GUS* repression, which became even more apparent in seedlings grown in liquid media. Subsequent analyses of liquid-grown *gin2.1* and *oe3.2* seedlings revealed that Glc -dependent regulation of the five selected genes was not affected in *gin2.1*, whereas it was enhanced in *oe3.2* plants for *bZIP63, At5g22920*, and *BT2*. The sugar treatments had no effect on ATP/ADP ratio, suggesting that changes in gene expression were not linked to cellular energy status. Overall, the data suggest that HXK1 does not act as Glc sensor controlling *bZIP63, At5g22920*, and *BT2* expression, but it is nevertheless required for the production of a downstream metabolic signal regulating their expression.

## Introduction

Within plant cells, carbohydrates (CH) undergo a constant production, transport and metabolization, including inter-conversion, polymerization and degradation. All these processes produce a multitude of different sugar molecules, which may act as specific signals to coordinate the metabolism and growth between cells producing and receiving sugars. In response to both environmental and developmental changes, total pool sizes of these potential signals are in constant fluctuation. This can be signaled by different sugar-specific pathways (reviewed in Smeekens, 2000; Hanson and Smeekens, 2009; Baena-González, 2010; Eveland and Jackson, 2011; Granot et al., 2013; Tognetti et al., 2013; Lastdrager et al., 2014) controlling, among other processes, the differential expression of sugar-responsive genes. This, in turn, leads to adaptation of the plant growth and development relative to the internal CH status.

Whole-genome expression studies on different plant systems have identified a multitude of genes responsive to the exogenous application of simple sugars, such as glucose (Glc), fructose (Fru) and/or sucrose (Suc) (Koch, 1996; Bläsing et al., 2005; Osuna et al., 2007; Kunz et al., 2014). The observed changes in sugar-responsive gene expression mimicked the transcriptional regulation in plants undergoing physiological fluctuations of sugar contents, e.g., during the diurnal cycle (Koch, 1996; Usadel et al., 2008; Kunz et al., 2014). The regulation of expression of responsive genes by exogenous and/or endogenous sugar(s) may to a large extent depend on the uptake capacity or transport, as well as the metabolization rate of the sugar by enzymes of the primary metabolism (Chaudhuri et al., 2008; Gout et al., 2011). Examples of such proteins include: (i) triose-phosphate-transporter (TPT; corresponding mutant *tpt2*), involved in carbon export from chloroplasts to the cytosol; (ii) hexokinase (HXK1; *gin2.1, oe3.2*), catalyzing the phosphorylation of Glc and Fru to provide substrate for glycolysis as well as Suc and starch synthesis (Granot et al., 2013); (iii) Suc synthase (SUS; *sus1-4, sus5/6*), catalyzing the breakdown of Suc to Fru and UDP-Glc, the latter being an essential precursor for oligo- and polysaccharides (Kleczkowski et al., 2010); as well as (iv) ADP-Glc-phyrophosphorylase (AGPase, *adg1*) and (v) glucan-water-dikinase (GWD, *sex1*) which are essential for synthesis and breakdown of starch, the pivotal CH/energy reserve (Caspar et al., 1991). Mutants impaired in the activity of one of these proteins (or several isozymes as in multiple *sus* mutants) have frequently exhibited unaffected growth phenotypes and minor changes in the overall internal content of soluble sugars under standard diurnal conditions (12/12 or 16/8 h light/dark photoperiod; light intensity ~150 μE) (Caspar et al., 1991; Moore et al., 2003; Barratt et al., 2009; Schmitz et al., 2012). This suggests that the affected pathway is likely compensated for by other convergent metabolic pathways, as suggested for SUS isozymes and cytosolic invertases (Barratt et al., 2009). Assuming that such a compensation might result in changes in the size and turnover of specific sugar-pools and lead to differential expression of certain sugar-responsive genes, these mutants represent a valuable tool to assess the relevance of the respective pathway in generation of the signaling molecule.

Recently, using an *Arabidopsis* cell culture system, we have identified 290 genes whose expression was rapidly affected by 1 mM Glc, Fru, or Suc (Kunz et al., 2014). About 20% of those genes were earlier identified as responsive to short time treatment of *Arabidopsis* seedlings with 15 mM Suc (Osuna et al., 2007). In both studies, the effects of external sugars on gene expression were accompanied by rapid metabolization of the sugars supplied (Osuna et al., 2007; Kunz et al., 2014). As short-term treatments with exogenous sugar cause fast perturbations of the sugar balance, overwhelming the interior and exterior of the cells with sugars (Chaudhuri et al., 2008; Gout et al., 2011), levels of other metabolically related compounds, possibly important for sugar-dependent signaling, might also be affected. Channeling of the imported Suc or Glc into the primary metabolism requires the action of HXK, an enzyme previously suggested to act both in Glc-metabolism and as a sensor in the Glc-signaling pathway (Jang et al., 1997; Moore et al., 2003). However, the Glc-sensing property of HXK does not apply ubiquitously to all cell types and under all growth and developmental conditions (Granot et al., 2013).

In the present work, we have selected five genes (*bZIP63, At5g22920, BT2, MGD2*, and *TPS9*), which were previously shown to be responsive to the exogenous supply of Glc, Fru, and Suc (Kunz et al., 2014). Even though the responsiveness could partly be related to a rapid metabolic interconversion between those sugars, a possible conversion of the applied sugars to other metabolic signal(s) could not be excluded. Here we show that, under physiological conditions, a number of proteins/enzymes that are involved in the CH-metabolism (HXK1, TPT2, AGPase, GWD, and several isozymes of SUS) are not essential for the generation of the signaling molecule that triggers sugar-dependent changes in gene expression of selected genes. However, under conditions which result in drastic short-term perturbations of the intracellular sugar homeostasis, we show that the metabolic function of the HXK1 is required to generate a signaling molecule which induces repression of *BT2, At5g22920*, and *bZIP63*.

## Materials and methods

### Plant material

Seedlings of the *Arabidopsis thaliana* wild types (wt) Colombia (Col-0), Landsberg (Ler-0), Bensheim (Be-0) and the loss-of-function mutants *adg1* (wt background Col-0, Caspar et al., 1991), *sex1* (Col-0, Ritte et al., 2002), *sus1*/*sus2*/*sus3*/*sus4* (Col-0, called *sus1-4*), and *sus5*/*sus6* (Col-0, called s*us5/6*) (Barratt et al., 2009), *tpt2-1* (Col-0, Schmitz et al., 2012) and *gin2.1* (Ler-0, Moore et al., 2003), the gain-of-function mutant *hxk1_oe3.2* (Be-0, referred to as *oe3.2*, Jang et al., 1997) as well as the *pbZIP63::GUS* reporter line (Col-0, Matiolli et al., 2011) were analyzed for expression patterns of selected genes under different growth and treatment conditions.

### Plant growth and treatment

The analysis of gene expression during the physiological fluctuation of the endogenous sugar availability was conducted using 13-day-old seedlings of wild type (wt) Col-0, Ler-0, Be-0, and the mutant genotypes *adg1, sex1, sus1-4*, s*us5/6, tpt2-1, gin2.1*, and *oe3.2* grown on 0.5× MS 1.4% (w/v) agar without added sugar in long day photoperiod (16 h light, 8 h dark; 150 μmol m^−2^ s^−2^). Seedlings were harvested at the end of the light period and at the end of a 6 h extended night by separating roots from shoots and immediately snap-freezing the material in liquid N_2_.

The effect of exogenous application of sugar on gene expression was tested in 8-day-old seedlings of Ler-0 wt, Be-0 wt, *gin2.1*, and *oe3.2* germinated and grown for 7 days in 0.5× liquid MS media supplemented with 30 mM Suc and 1 mM MES (pH 5.6, adjusted with KOH). Seedlings were incubated on an orbital shaker (121 rpm) in long day conditions (16 h light, 8 h dark, 150 μmol m^−2^ s^−2^). Prior to the exogenous application of either 1% Suc or Glc for 1 h, the growth medium was replaced with new 0.5× MS without any sugar supplement, followed by a starvation period of 24 h in the dark. Subsequently, seedlings were harvested by brief washing using 0.5× MS without sugar, dried with paper towel and snap-frozen in liquid N_2_.

Effects of different growth conditions and exogenous sugar application were studied using the *pbZIP63::GUS* reporter line. The seedlings were assayed for *GUS*-expression after growth for 7 days (16 h light, 8 h dark, 150 μmol m^−2^ s^−2^) on 0.5× MS 1.4% (w/v) agar supplemented either with or without sugar (0.3% or 3% Glc or Suc). In addition, seedlings were grown for 7 days (16 h light, 8 h dark, 25 or 150 μmol m^−2^ s^−2^) in 0.5× liquid MS media supplemented with 30 mM Suc and 1 mM MES (pH 5.6, adjusted with KOH). After replacement of the growth media with 0.5× MS without sugar and a 24 h starvation period in the dark, the seedlings were treated for 6 h with 1% Suc or Glc and subsequently assayed for *GUS*-expression.

### RNA extraction and cDNA synthesis

Total RNA from seedlings was isolated by using the E.Z.N.A® Plant RNA kit (R6827-01, OMEGA BioTek). Following elution of RNAs, DNA contaminations were removed using DNase treatment with the DNA-*free*™ DNase treatment and removal kit (AM1906, Ambion®). cDNAs were generated using SuperScript™ II Reverse Transcriptase (Invitrogen) and Oligo(dT)_18_ primers (Fermentas Life Science) following the manufacturers protocol. 0.5 μg total RNA was used in each reaction.

### Gene expression analyses

Quantitative real-time PCR was conducted using the Roche LightCycler®480 Real-Time PCR system and the Roche LightCycler®480 SYBR Green I Master following the manufacturer's protocol. Sequences of RT-PCR primers for all tested genes are listed in Table S1. *PP2A* was used as endogenous reference gene (Czechowski et al., 2005). Its stable expression in *A. thaliana* was confirmed for sugar-treated plant material using the Genevestigator expression analysis tool (Hruz et al., 2008). To assess the statistical significance of differences in gene expression between the various treatments, a Student's *t*-test (two-tailed distribution, unequal variance) was applied.

### Carbohydrate analyses

Frozen plant material was powdered in liquid N_2_ using a cooled mortar and pestle. The soluble sugars Suc, Glc, and Fru were extracted from the samples using an ethanol-water based buffer and the internal sugar contents were measured using a NADP-coupled enzyme assay as described in Kunz et al. (2014).

### GUS staining

Whole seedlings of the *pbZIP63::GUS* reporter line were transferred into GUS-staining solution (1 mM X-Glc, from Fermentas/ Thermo Scientific); 50 mM NaHPO_4_, pH 7.0; 1% v/v TritonX-100) following the different growth conditions and treatments described above. The samples were incubated for a maximum of 1.5 h at 37°C in the dark. Afterwards the samples were de-stained in 90% ethanol for several hours and finally stored in 50% sterile glycerol. The samples were analyzed and results documented by using a Zeiss Axioplan 2 microscope and a Canon EOS 650D.

### ATP/ADP measurements

Liquid-media-grown plants were snap-frozen with liquid N_2_ and subsequently ground in liquid N_2_ to a fine powder. Adenylates were extracted by addition of 1 ml of ice-cold 3% TCA to 25 mg of plant powder. After subsequent centrifugation for 2 min, the supernatant was stored at −20°C for further analysis. ATP and ADP contents were assayed by the firefly luciferase method using LKB 1250 (Wallac) luminometer, as described earlier (Gardeström and Wigge, 1988).

## Results

### Impact of diurnal changes in sugar contents on sugar-responsive gene expression under steady growth conditions

In order to investigate the relevance of distinct CH-metabolism pathways for the production of sugar signal(s), we analyzed mutants impaired in Suc metabolism (*sus1-4* and *sus5/6*), triose-P transport (*tpt2*), and starch biosynthesis (*adg1*) and degradation (*sex1*) to assess their impact on sugar-responsive gene expression. Changes in soluble sugar contents and the expression of the previously identified sugar-responsive genes *bZIP63, At5g22920, BT2, MGD2*, and *TPS9* (Kunz et al., 2014) were studied in shoot and root tissues of agar-grown 13-day-old *A. thaliana* wt and mutant seedlings harvested at the end of the day and the end of a 6 h-extended night. The samples represented states of high (end of day) and low (end of night) internal sugar contents for both heterotrophic (roots) and autotrophic (shoots) tissues.

**Figure 1:**
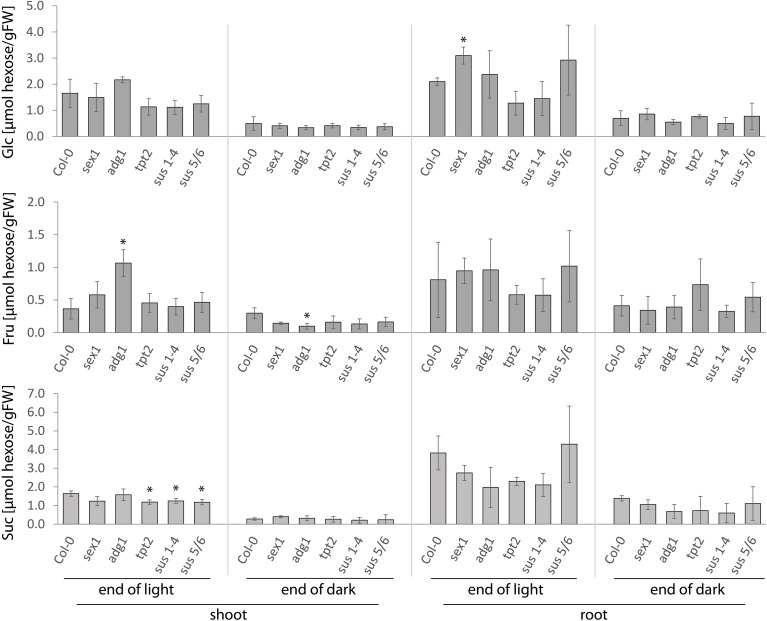
**Internal concentrations of Glc, Fru, and Suc in shoots and roots of *A. thaliana* wt plants and mutants impaired in CH-metabolism**. Plants were grown for 13 days on agar under 16 h light/ 8 h dark photoperiod, and the samples were taken both at the end of day and the end of a 6 h extended night (14 h dark). Significance: *t*-test; ^*^α = 0.05, *n* = 3.

As expected, in all studied genotypes a decrease in contents of soluble sugars was observed between the end of the day and the end of a 6 h-extended night (Figure 1). However, this decrease was hardly affected in the mutant backgrounds. As exceptions, mutants impaired in Suc metabolism (*sus1-4* and *sus5/6*) and triose-phosphate transport (*tpt2*) showed a small decrease in Suc content in shoots at the end of light conditions, while starch mutants exhibited altered Fru content in shoots (*adg1*, both in light and dark) and an increase in Glc in roots (*sex1*, end of light) (Figure 1). The differences observed between wt and mutant lines were relatively small, with at best a two-fold increase in Fru content between control and *adg1* for shoots harvested at end of night. Generally, the results underline the flexibility of plants to bypass CH-associated mutations and maintain an overall CH balance. This, however, does not exclude the possibility that the distribution of sugars between different subcellular pools is affected in the mutants.

**Figure 2:**
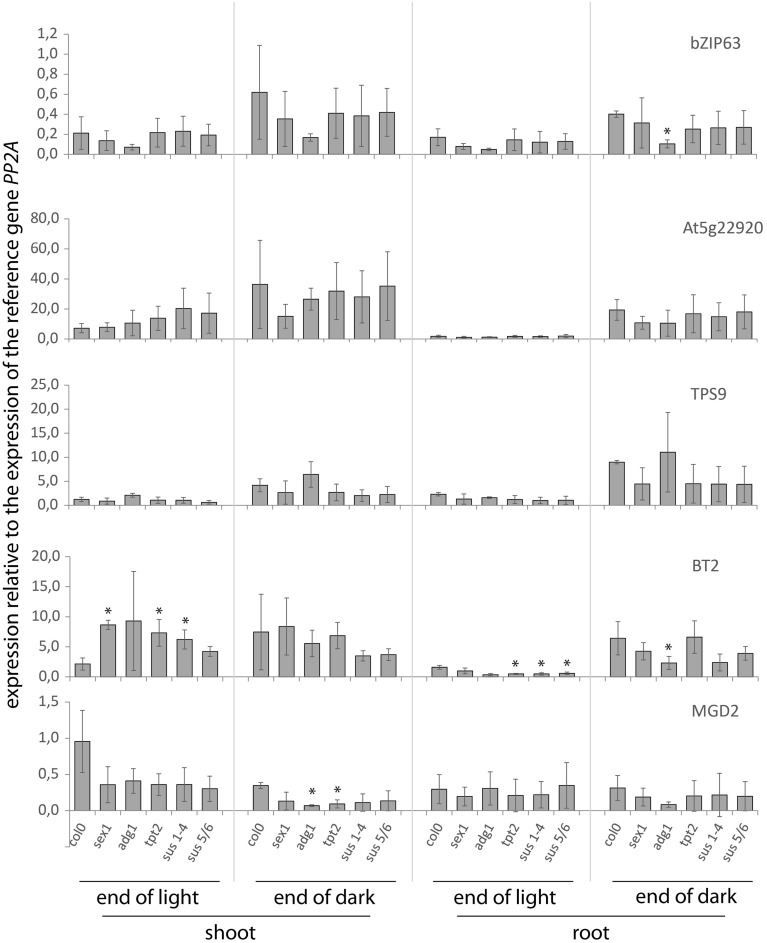
**Expression of selected genes in shoots and roots of *A. thaliana* wt plants and mutants impaired in CH-metabolism**. Plants were grown for 13 days on agar under 16 h light/ 8 h dark photoperiod, and the samples were taken both at the end of day and the end of a 6 h extended night (14 h dark). The data represent the ratio of a given gene expression to the expression of the reference gene (*PP2A*). Significance: *t*-test; ^*^α = 0.05, *n* = 3.

The wt expression of the five selected genes followed the overall sugar availability both in shoot and root tissues, with *MGD2* expression being induced at the end of the day, concomitantly with the increase in internal sugar contents (Figures 1, 2), and the expression of the other genes being repressed under the same conditions. Overall, transcriptional regulation of the selected genes led to ca. two to five-fold changes in expression between light and dark conditions. Gene expression profiles in the mutants were comparable to those found in wt plants, with the notable exception of *BT2* and, to some extent, *bZIP63* and *MGD2* expression.

The *BT2* expression was significantly higher in shoots of *tpt2, sus1-4*, and *sex1* mutants harvested at the end of the day (Figure 2). This increase correlated with a slight, though significant, decrease of internal Suc levels in *tpt2* and *sus1-4* (but not in *sex1*) samples (Figure 1), and was consistent with earlier data that *BT2* expression was Suc-responsive (Kunz et al., 2014). In comparison to the wt, there was also a significant repression of *BT2* expression in roots of *tpt2, sus1-4*, and *sus5*/*6* harvested at the end of the day (Figure 2), but this was not accompanied by significant differences in sugar content in those mutants (Figure 1). Both *BT2* and *bZIP63* expression was significantly decreased in roots of the *adg1* mutant at the end of the extended dark period (Figure 2), but again this was not accompanied by changes in soluble sugars (Figure 1). *MGD2* showed a lower expression in darkened shoots of *adg1* and *tpt2* (Figure 2), which in the case of *adg1* plants could perhaps be related to a decreased Fru content (Figure 1).

To obtain an integrated view of the sugar-dependency of the genes studied, diurnal changes in gene expression in both wt and the mutants were directly compared to corresponding changes in sugar content, using data points from Figures 1, 2 (Figure S1). When plotted in this way, sugar-stimulated *MGD2* gene exhibited a logarithmic correlation with sugar content, while sugar-repressed genes showed an exponential correlation (Figure S1). This indicated that all tested genes responded to changes in sugar content, independently of the mutant background and organ considered. R^2^ correlation factors varied between gene, sugar and organ studied: expression of *bZIP63* showed best correlation with Glc rather than other sugars in both shoots and roots, *At5g22920* showed best correlation with Glc in roots but with Suc in shoots, while expression of *TPS9* was correlated with Glc and Suc in both organs. On the other hand, expression of *BT2* was correlated with sugars in roots, but not in shoots. Compared to other genes, *MGD2* showed the weakest correlation to any specific sugar in both roots and shoots. The results (Figure S1) confirmed that the genes tested were sugar-responsive, but no specific sugar signal could be preferentially associated/excluded, based on those studies.

Overall, the results with mutants impaired in CH synthesis/metabolism/transport were inconclusive as to the nature of sugar signal(s) regulating expression of the selected genes. The long-term adaptation of CH metabolism in these mutants, reflected by generally wt-like soluble sugar contents, makes it difficult to identify such a signal or its precise origin. Also, a possibility cannot be excluded that the actual signal derives from an intermediate of sugar metabolism rather than the sugar itself.

### HXK1 is not essential for transcriptional regulation of selected genes under steady growth conditions

To assess the importance of HXK1 for sugar-induced transcriptional regulation under physiological conditions, expression profiles of the selected genes were analyzed in shoot tissue of an *A. thaliana HXK1* loss- (*gin2.1*) and gain-of-function (*oe3.2*) lines. Both lines have previously been used to test the dependency of Glc-responsive gene expression on the Glc-sensing activity of HXK1 (Jang et al., 1997; Ciereszko et al., 2001; Ciereszko and Kleczkowski, 2002a,b; Moore et al., 2003; Karthikeyan et al., 2007); for instance, identifying CAB (*At3g27690*) as the marker gene for HXK-sensed Glc-signaling (Jang et al., 1997; Moore et al., 2003). Shoots from 13-day-old agar-grown transgenic plants and their respective wt ecotypes (Ler-0 for *gin2.1*, and Be-0 for *oe3.2*) were harvested at the end of the day and after a 6 h extended night.

**Figure 3:**
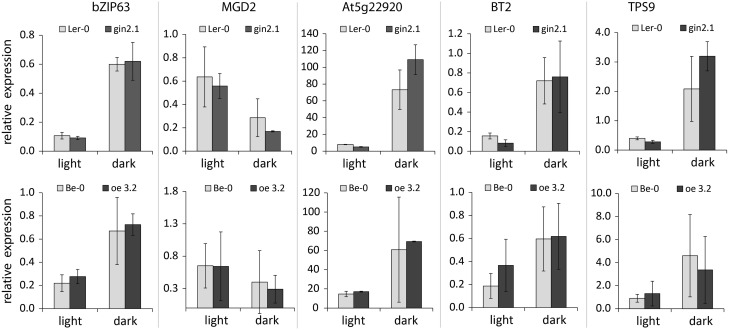
**Expression of selected genes in shoots of *A. thaliana* wt plants and *HXK1*- transgenics (*gin2.1* mutant and *oe3.2* overexpressor)**. Plants were grown for 13 days on agar under 16 h light/ 8 h dark photoperiod, and the samples were taken both at the end of day and the end of a 6 h extended night. The data represent the ratio of a given gene expression to the expression of the reference gene (*PP2A*). Significance: *t*-test.

Similar to the results with CH metabolism mutants (Figures 1, 2), the diurnal transition led to changes in expression for all five genes studied (Figure 3). However, no significant differences in expression were observed between wt controls and *HXK1*-transgenic lines for any of the genes, suggesting that, under the steady-state growth conditions applied, HXK1 is not essential for regulation of the selected genes. Assuming that *HXK1*-transgenic plants, similar to other mutants in CH metabolism (Figure 1), undergo adaptation to restore CH homeostasis, the lack of differential expression may have resulted from metabolic compensation of the effect of the HXK1 deletion/overexpression. This suggested that, in order to observe a putative HXK-dependent regulation of sugar-responsive gene expression, plants should be submitted to a sudden rapid increase of the content of a putative signaling molecule, e.g., in response to an exogenous sugar application (Chaudhuri et al., 2008; Gout et al., 2011).

### Experimental system to assess short-term sugar effects on gene expression in whole plants

**Figure 4:**
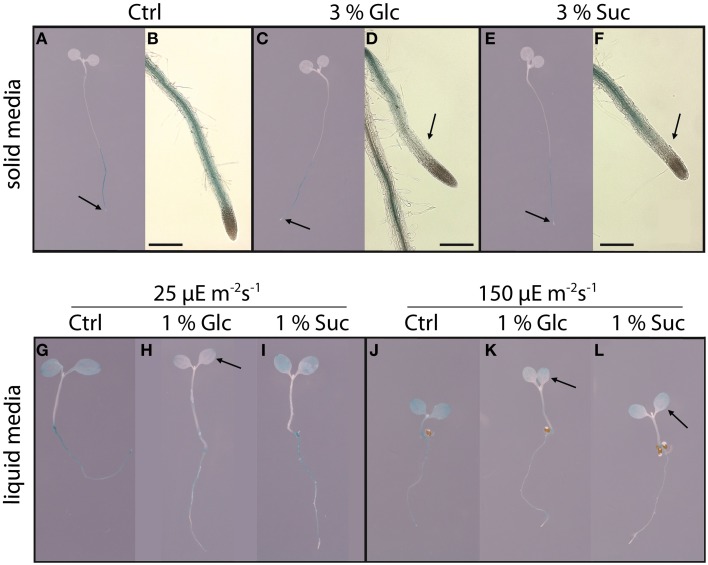
**Effects of growth conditions on sugar-dependent expression of *bZIP63* in the *pbZIP63::GUS* line**. The whole *A. thaliana* seedlings were grown for 7 days under a 16 h light (either 25 or 150 μmol m^−2^ s^−1^)/8 h dark photoperiod either on agar **(A–F)** or in liquid media **(G–L)**. Following a 24 h dark-treatment, the seedlings were dipped for 6 h either into water (controls **A,B,G,J**), 3% Glc **(C,D)**, 3% Suc **(E,F)**, 1% Glc **(H,K)**, or 1% Suc **(I,L)**, and the expression of *bZIP63* was visualized by GUS staining. Plants shown in panels **(A–F)** were grown under 150 μmol m^−2^ s^−1^ light in 16 h/8 h photoperiod. Size of the scale bar is 500 μM. Each figure shows a typical example from three repeats conducted within an interval of 2 weeks. Each repeat contained 3–5 seedlings. Black arrows indicate the areas in which the GUS positive labeling is diminished in response to external sugar treatment.

To define conditions under which the application of exogenous sugars rapidly and strongly affects the expression of sugar-responsive genes, we used a previously described *pbZIP63::GUS* reporter line (Matiolli et al., 2011). In seedlings of *A. thaliana* which were grown on solid 0.5× MS media, expression of *pbZIP63::GUS* was restricted to the root tissue (Figure S2), as earlier reported (Weltmeier et al., 2009; Kunz et al., 2014). Supplementing the medium with 0.3 or 3% of either Glc or Suc had no effect on the expression in any part of the seedling (Figure S2). These observations confirmed that the continuous supply of sugar through the media did not impair the establishment of a steady-state CH-balance during germination and seedling growth, and resulted in a comparable *pbZIP63::GUS* expression in both control and treated samples. Subsequently, using *pbZIP63::GUS* expression as a proxy reflecting destabilized cellular CH-balance, seedlings were first grown on solid 0.5× MS media without added sugar, and then dipped for 6 h into solutions with either 3% Glc or Suc. This resulted in a visible decrease of *pbZIP63::GUS* expression only in the elongation zone of the roots (Figures 4A–F). Even though the complete seedlings were submerged in the sugar treatment solution, no changes in *GUS* expression were observed in cotyledons, possibly due to the already low GUS activity in this organ prior to sugar application (Figure 4A).

In contrast to the lack of *pbZIP63::GUS* expression in cotyledons of seedlings grown on solid MS media, the germination and growth of seedlings in liquid 0.5× MS did induce *pbZIP63::GUS* expression in both roots and cotyledons (Figures 4A,G,J). GUS activity in roots appeared slightly reduced, when compared to solid media-grown plants (Figures 4A,G,J), and there were also some differences in GUS staining between normal (150 μE m^−2^ s^−1^) compared to dim (25 μE m^−2^ s^−1^) light conditions (Figures 4G,J). Using the liquid-grown seedlings, a strong and rapid response to the external sugar supply was observed by the down-regulation of *pbZIP63::GUS* expression in seedlings exposed for 6 h to 1% of either Glc or Suc (Figures 4H,I,K,L). This repression, which was independent of the light conditions, was strongest in Glc-treated samples and was observed in both cotyledons and roots. The results indicated that the treatment of *A. thaliana* seedlings using liquid-based growth conditions led to prompt and strong changes in gene expression upon application of exogenous sugars. Moreover, the change from solid to liquid growth conditions expanded the distribution of expression to new tissue/organ (cotyledons), which was directly in contact with the surrounding medium. Since the liquid-media treatment proved to be suitable to trigger strong sugar-dependent *bZIP63* expression, this system was used in further studies on the involvement of HXK1 in sugar-dependent expression of selected genes.

### Metabolic function of HXK1 is required for regulation of *bZIP63, At5g22920*, and *BT2* expression by exogenous sugars

**Figure 5:**
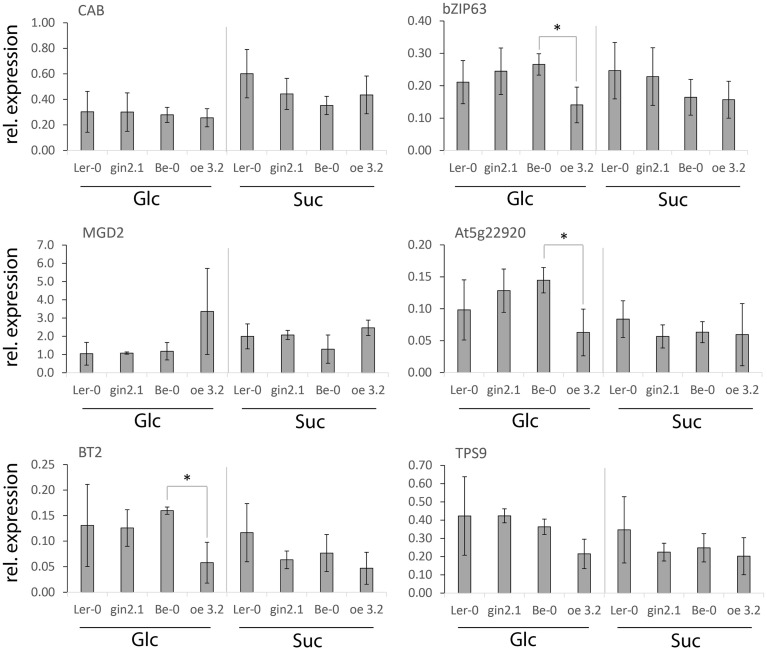
**Effects of short (1 h) exposure to external Glc or Suc on the expression of selected genes in shoots of *A. thaliana* wt plants and *HXK1*-transgenics (*gin2.1* mutant and *oe3.2* overexpressor)**. The *A. thaliana* seedlings were grown for 7 days in liquid media under a 16 h light/8 h dark photoperiod, followed by a 24 h dark treatment. Subsequently the seedlings were dipped for 1 h either into water, 1% Glc or 1% Suc, and the samples were collected for qPCR. The data represent the relative gene expression normalized to the control-treatment (water). Data are shown as average ± sd. Significance: *t*-test; ^*^α = 0.05, *n* = 3.

Liquid-media-grown seedlings of *gin2.1, oe3.2* and their respective controls were treated for 1 h with 1% Glc or Suc and were subsequently analyzed for expression of the five selected genes. Irrespective of the genotype, *MGD2* expression showed only little or no response to the exogenous application of either Glc or Suc (Figure 5). In contrast, changes in expression of other genes upon Glc- and Suc- treatments ranged from ca. two to three fold (*TPS9* and *CAB*) to three to five fold (*bZIP63*) to 10–20 fold (*At5g22920, BT2*) (Figure 5). While repression of *bZIP63, At5g22920*, and *BT2* was comparable between Ler-0 wt and *gin2.1*, it was significantly enhanced upon Glc-treatment of the *oe3.2* seedlings. The Glc-dependent enhancement of gene repression in *oe3.2* was also observed for *TPS9*, but the difference to the wt was not significant. Regardless of the treatment, expression of *MGD2* and *CAB* was similar between the transgenics and their respective wt plants (Figure 5), indicating that under the applied conditions HXK1-dependent Glc-sensing was ineffective.

Taken together, the results suggest that Suc- and/or Glc-dependent mechanisms influence the transcriptional regulation of *bZIP63, At5g22920*, and *BT2* upon exogenous sugar-treatment in *A. thaliana* seedlings. While the experimental conditions apparently limit the Glc-sensing function of the HXK1, as shown by stable expression of *CAB*, it can be assumed that regulation of the repression of *bZIP63, At5g22920*, and *BT2*, which is enhanced in the *oe3.2* plants (Figure 5), requires an involvement of the metabolic function of HXK1 for the generation of the signal.

### Sugar accumulation and energy status in HXK1 transgenic plants supplied with exogenous sugars

**Figure 6:**
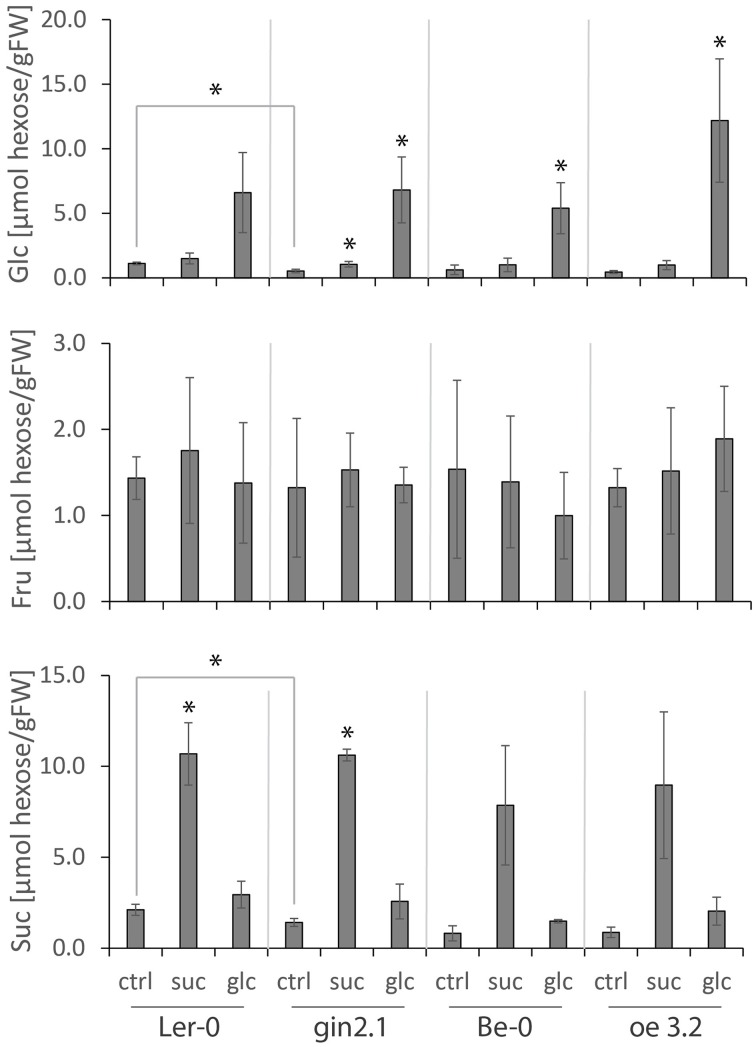
**Effects of short (1 h) exposure to external Glc or Suc on internal sugar contents in shoots of *A. thaliana* wt plants and *HXK1*-transgenics (*gin2.1* mutant and *oe3.2* overexpressor)**. The *A. thaliana* seedlings were grown for 7 days in liquid media under a 16 h light/8 h dark photoperiod, followed by a 24 h dark treatment. Subsequently the seedlings were dipped for 1 h either into water, 1% Glc or 1% Suc, and the samples were collected for sugar content analyses. The star indicates significant difference either between untreated wt and *gin2.1* mutant or between untreated and treated samples. Significance: *t*-test; ^*^α = 0.05, *n* = 3.

The utilization of imported sugar in the *HXK1*-gain-of function mutant *oe3.2* could result in an increased metabolic flux of carbon through the HXK reaction, providing more precursors for e.g., glycolysis, Suc- and starch-synthesis, and thereby influencing the availability of energy in the form of ATP. However, when comparing *oe3.2* and control (Be-0) wt plants, the observed enhanced repression of *bZIP63, At5g22920*, and *BT2* in *oe3.2* was not accompanied by significant differences in internal sugar concentration (Figure 6). The Suc-treated samples showed some non-significant increase in Glc-content within 1 h, similar to the slight increase of Suc upon Glc-provision, which suggests limited inter-conversions between the sugars applied. The Fru-content similarly remained stable after the treatment. Even though Glc- and Suc-contents in *gin2.1* were significantly decreased compared to control (Ler-0) wt in untreated samples (Figure 6), the internal sugar content in response to the treatment was comparable between the transgenic and control plants (Figure 6).

When the data for internal sugar contents in HXK1 transgenic plants and their controls were directly plotted against the expression of genes under study (Figure S3), the relationship between sugar content and gene response roughly followed “standard curves” derived from data in Figures 1, 2. In wt and *gin2.1* plants, the patterns of *bZIP63, At5g22920*, and *BT2* expression were similarly independent of the sugar considered (Glc or Suc), whereas *oe3.2* overexpressor showed an enhanced response (when compared to its Be-0 wt control) only in relation to Glc, but not Suc (Figure S3). Although a similar pattern was observed for *TPS9* expression, the data points for expression of this gene at high sugar concentration were outside its “standard curve” for sugar response. A similar shift from “standard curve” was observed for *MGD2*, where effects of both Glc and Suc were enhanced in *oe3.2* plants (Figure S3). Thus, under conditions of a rapid exposure to higher sugar content, the expression of *TPS9* and *MGD2* partly uncoupled from the “standard curves,” suggesting additional mechanism(s) of regulation.

**Figure 7:**
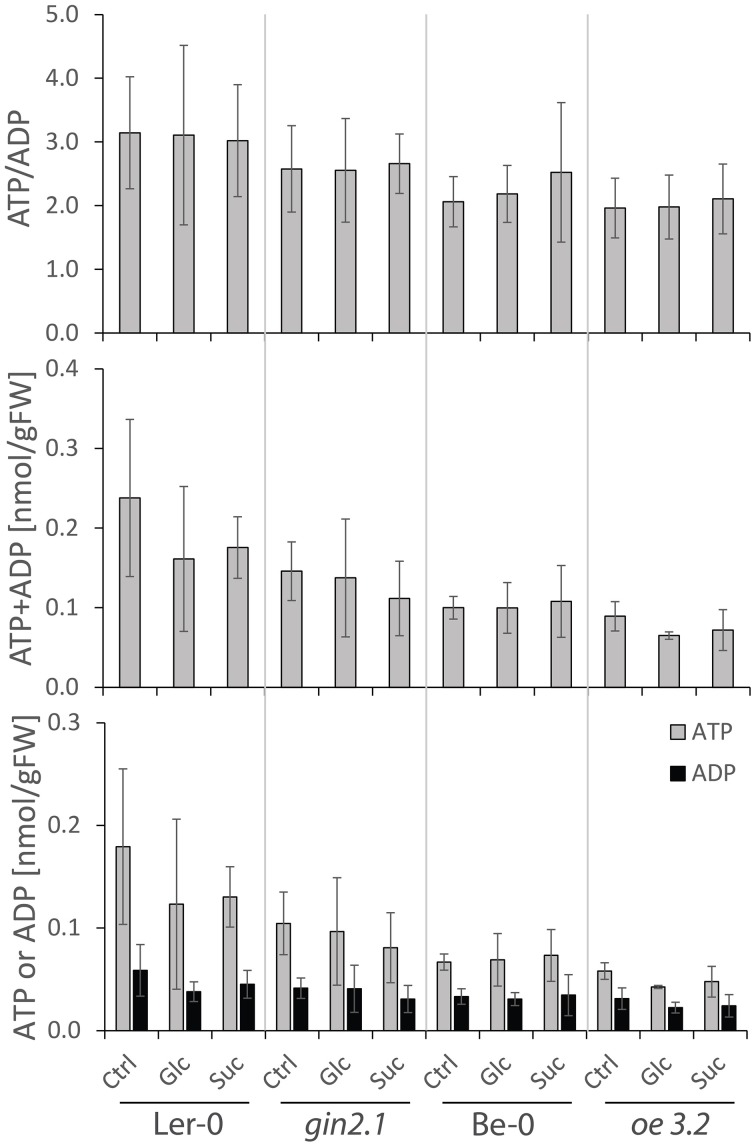
**Effects of short (1 h) exposure to external Glc or Suc on ATP and ADP contents in shoots of *A. thaliana* wt plants and *HXK1*-transgenics (*gin2.1* mutant and *oe3.2* overexpressor)**. The *A. thaliana* seedlings were grown for 7 days in liquid media under a 16 h light/ 8 h dark photoperiod, followed by a 24 h treatment. Subsequently, the seedlings were dipped for 1 h either into water, 1% Glc or 1% Suc, and the samples were collected for adenylate content analyses. Data are shown as average ± sd. Significance: *t*-test.

To assess whether exogenous sugars had any effect on the energy status in *HXK1*-transgenic plants, we analyzed ATP and ADP contents in both *gin2.1* and *oe3.2* and their respective wt plants. The seedlings, after being treated with 1% Glc or Suc for 1 h, exhibited comparable total pools of ATP and ADP and similar ATP/ADP ratios, independently of the treatment or the genotype (Figure 7). This indicated that production and consumption of energy equivalents promptly adjusted to the increased sugar availability. As a consequence, it seems plausible to assume that the generation of a signal (or signals) triggering changes in expression of *bZIP63, At5g22920*, and *BT2* was independent of the energy gain derived from the applied sugar, but required the activity of the HXK1. More studies involving assays of ATP, ADP (and AMP) in different cellular compartments would be required to test in detail cell energetics in plants with modified HXK1 expression.

## Discussion

Distribution of the primary CH-metabolism pathways among different compartments leads to a spatial distribution of metabolites which may act as putative signals for gene regulation. Even though the sugar-dependent expression of the nuclear-encoded genes *bZIP63, At5g22920, MGD2, BT2*, and *TPS9* is likely to be regulated through a cytosolic mechanism, it cannot be excluded that the generation of the signal is either localized in another compartment or depends on intermediate(s) produced in other parts of the cell. Here we show that, under physiological conditions, the expression of *BT2* is impaired in *A. thaliana* seedlings mutated in the *TPT* and *SUS* genes (Figure 2). Both TPT and SUS activities are essential for Suc-metabolism and, thus, for allocation of carbon for e.g., respiration, starch-, cellulose- and cell wall- production in both sink and source tissues (Bieniawska et al., 2007; Barratt et al., 2011; Baroja-Fernández et al., 2012). Similar to the suggestion that the mutation of *TPT* (*tpt2* mutant) is compensated by Glc-export from the chloroplast to the cytosol via starch metabolism (Schneider et al., 2002; Schmitz et al., 2012), it has been shown that *sus1-4* mutant has an enhanced invertase-dependent Suc-cleavage pathway in the cytosol (Barratt et al., 2009), resulting in wt-like phenotypes for both mutants. The altered Suc-content in the illuminated shoots of *tpt2* and *sus1-4* mutants (Figure 1) suggests that *BT2* expression is determined by cytosolic Suc-availability. This goes along with the postulated Suc-dependent bZIP11-mediated regulation of *BT2* expression (Hanson et al., 2008) and with effects of the Suc-analog turanose on *BT2* expression in *A. thaliana* cells (Kunz et al., 2014). Since several mutants of proteins known to be involved in primary CH-metabolism maintained balanced CH-pools under the steady growth conditions applied, the regulation of expression of other sugar-responsive genes (*bZIP63, At5g22920, MGD2*, and *TPS9*) was not altered when compared to wt plants (Figures 2, 3).

Given the fact that sugar-resupply to carbon-deprived *A. thaliana* cells and seedlings results in a fast increase in the cytosolic hexose and hexose-P pools (Osuna et al., 2007; Gout et al., 2011), it can be assumed that upon the import of the applied sugar into the cytosol it will be quickly phosphorylated by HXK to be fed into the CH-metabolism and support the growth-recovery. In this study we showed that, compared to the unaltered expression of *MGD2* and *TPS9* between transgenic and wt plants, the Glc-dependent repression of *bZIP63, At5g22920*, and *BT2* was unaffected by the loss of the HXK1 Glc-sensing activity in the *gin2.1* mutant, but was enhanced in the *oe3.2* (Figure 5). A similar mechanism, involving signaling by a downstream HXK metabolite, was earlier suggested for certain pathogenesis related (*PR*) genes, based on their enhanced expression in HXK overexpressing plants (Xiao et al., 2000). Both the signaling compound and a sensor for this signal are unknown at present. In addition to the Glc-dependent regulation requiring the HXK-pathway, shoot *BT2* expression appears also to be Suc-sensitive, whereas in roots it is sensitive to signal(s) arising from plant adaptation to *tpt2, sus, adg1*, and *sex1* backgrounds (Figures 1, 2). Whether those signals correspond to the same downstream metabolite of the HXK pathway, as suggested using *HXK1*-gain-of-function mutant (Figure 5), is unknown at present.

Previously it has been shown that bZIP63 mediates Kin10/SnRK1-signaling in the induction of reporter genes (Baena-González et al., 2007) and that the *At5g22920* and *BT2* expression is regulated via a bZIP11-mediated SnRK1-dependent pathway (Delatte et al., 2011), thereby implicating those genes in the cellular response to a sugar- and energy-deprivation status. On the other hand, our results on adenylate status in sugar-treated *HXK1*-overexpressor and wt plants (Figure 7) suggest that the sugar-dependent regulation of *bZIP63* as well as *At5g22920* and *BT2* expression is not linked to an alteration of the energy balance. In addition, it can be concluded that HXK1 is involved in the formation of a metabolic signal (or signals) which regulates the genes tested. Such a signal can be Glc-6P, the product of HXK reaction, or metabolites downstream from Glc-6P. With respect to G6P, given its important role in primary metabolism, large fluctuations in G6P content, unless very localized, could compromise its metabolic function. Trehalose-6-P (T6P), a downstream-intermediate of G6P and UDP-Glc metabolism, appears to be a better candidate to serve as signal, especially that its role in gene regulation is well documented (Nunes et al., 2013). On the other hand, T6P accumulation has been shown to be correlated with Suc-accumulation (Lunn et al., 2006), whereas in our study Suc-treatment did not lead to an enhanced repression of the genes under investigation (Figure 5). More studies, involving assays of G6P, T6P and other metabolites downstream from HXK reaction, are necessary to pinpoint the exact signal regulating genes of our interest. In any case, based on our data, HXK might act in the provision of the signal for SnRK1, mediating the adjustment of metabolism and growth.

In our previous work, using *A. thaliana* habituated cell culture, we suggested that Glc-signaling via HXK was involved in sugar-dependent expression of *bZIP63, At5g22920*, and *MGD2* (Kunz et al., 2014). This was based on effects of 2-deoxyglucose (2Dog), an analog of D-Glc, which is phosphorylated by HXK (Klein and Stitt, 1998). Here, using whole seedlings, we have confirmed the signaling role of HXK1 for *bZIP63* and *At5g22920*, and found that the signal comes downstream from HXK reaction. We have also shown that *BT2* is regulated by a similar mechanism (Figure 5). Whereas HXK1 is involved in Glc-signaling for those genes, we were unable (under experimental conditions used) to demonstrate its sensing function, which does not involve HXK catalytic activity (Moore et al., 2003). This was based on the fact that Glc-dependent expression of *CAB*, a marker for HXK1 sensing (Moore et al., 2003), was not affected in *gin2.1* and *oe3.2* transgenic plants (Figure 5). Much of the evidence in favor of the Glc-sensing function of HXK1 was obtained by a prolonged exposure of *HXK1*-transgenic plants or mesophyll protoplasts to high Glc concentrations (3–6%) (Jang et al., 1997; Xiao et al., 2000), whereas we used only short (1 h) treatments with 1% Glc or Suc (Figure 5).

## Conclusions

In this study, we analyzed the expression patterns of the sugar-responsive genes *bZIP63, At5g22920, TPS9, BT2*, and *MGD2* in mutants with altered CH-metabolism and/or signaling under physiological conditions as well as upon exogenous application of Glc and Suc. From our analyses, we can summarize the following major results: (i) In mutants impaired in the CH-metabolism, which were grown on solid media under physiological steady growth conditions, the dysfunctional pathways in Suc-, Glc-, and/or starch-metabolism were generally compensated for, thereby leading to wt-like soluble sugar content in the mutants; (ii) In those experiments, there was no clear indication as to the nature of a signaling compound (or compounds) regulating expression of the genes studied, except for possible effects of Suc on *BT2* expression; (iii) Growth of *A. thaliana* seedlings in liquid media expanded sugar-dependent *bZIP63* expression to aerial tissues; and (iv) The Glc-dependent repression of *bZiP63, At5g22920*, and *BT2* was independent of HXK1 sensing function, but was responsive to overexpression of HXK1, probably via some downstream product of HXK activity which served as a signal for gene regulation. This repression was independent of the soluble sugar accumulation as well as the ATP/ADP ratio, as shown in ectopic gain-of-function mutants of *HXK1* (*oe3.2*). The results with loss-of-function mutant of *HXK1 (gin2.1)* did not show any significant differences from those obtained with wt plants, possibly due to growth conditions applied. Altogether, our results suggest that the HXK is necessary to produce metabolic signal(s) regulating the three sugar-responsive genes. This was nevertheless only seen in response to relatively large and sudden changes of sugar content.

## Conflict of interest statement

The authors declare that the research was conducted in the absence of any commercial or financial relationships that could be construed as a potential conflict of interest.

## Supplementary material

Figure S1 **A relationship between internal Glc, Fru, and Suc content and expression of selected genes in solid-medium-grown *Arabidopsis* plants under steady-state conditions**. Standard response curves between sugar content (Glc in blue, Fru in red, and Suc in green) and gene expression were drawn for roots (left column) and shoots (right column) of 13 days old *A. thaliana* wt and mutants impaired in CH-metabolism at the end of the day and the end of a 6 h extended night. Genes monitored included bZIP63 **(A)**, MGD2 **(B)**, BT2 **(C)**, TPS9 **(D)**, and At5g22920 **(E)**. Correlation coefficients were given for each sample between a specific sugar variation and gene expression. The data were collected from Figures 1, 2.

Figure S2 **Effects of sugars added to the agar growth medium on expression of *bZIP63* in the *pbZIP63::GUS* line**. *A. thaliana* seedlings were germinated and grown (under a 16 h light/8 h dark photoperiod) for 7 days on solid 0.5× MS agar media containing either no sugar, or Glc (0.3 or 3%) or Suc (0.3 or 3%), and the expression of *bZIP63* was visualized by GUS staining.

Figure S3 **A relationship between internal Glc and Suc content and expression of selected genes in liquid-media-grown *Arabidopsis* wt plants and HXK1-transgenics (*gin2.1* mutant and *oe3.2* overexpressor) exposed to external sugar**. The plots were generated based on data from Figures 1, 2, 5, 6. Data points indicated by gray circles and spots represent “standard samples” and “standard curves,” respectively, and show the correlation between the averaged sugar and gene expression data from plants grown on solid media under steady state condition. The underlying data are derived from Figures 1, 2. Data points indicated by black, red and blue marks show the relation between sugar content and gene expression in wt and HXK1-transgenics. The underlying data are derived from Figures 5, 6. Please note that, for genes down-regulated by sugars **(A–D)**, the expression was clearly responsive to both Glc and Suc; however, it was only Glc, but not Suc, which at high concentration elicited an enhanced response in *oe3.2* overexpressor (when compared to its Be-0 wt control). For *MGD2* **(E)**, the expression was less sensitive to sugar content, with the exception of *oe3.2* plants where effects of both Glc and Suc were enhanced. Please note also that, at high sugar concentration, the data for *TPS9* and *MGD2* are considerably shifted from their respective “standard curves.”

Supplimentary Table 1 **List of qPCR primers**.
