# Supplementary figures and images for: Hexokinase 1 is required for glucose-induced repression of bZIP63, At5g22920, and BT2 in Arabidopsis
Source: Front Plant Sci. 2015 Jul 14;6:525. doi: 10.3389/fpls.2015.00525 (PMC4500909; doi:10.3389/fpls.2015.00525)

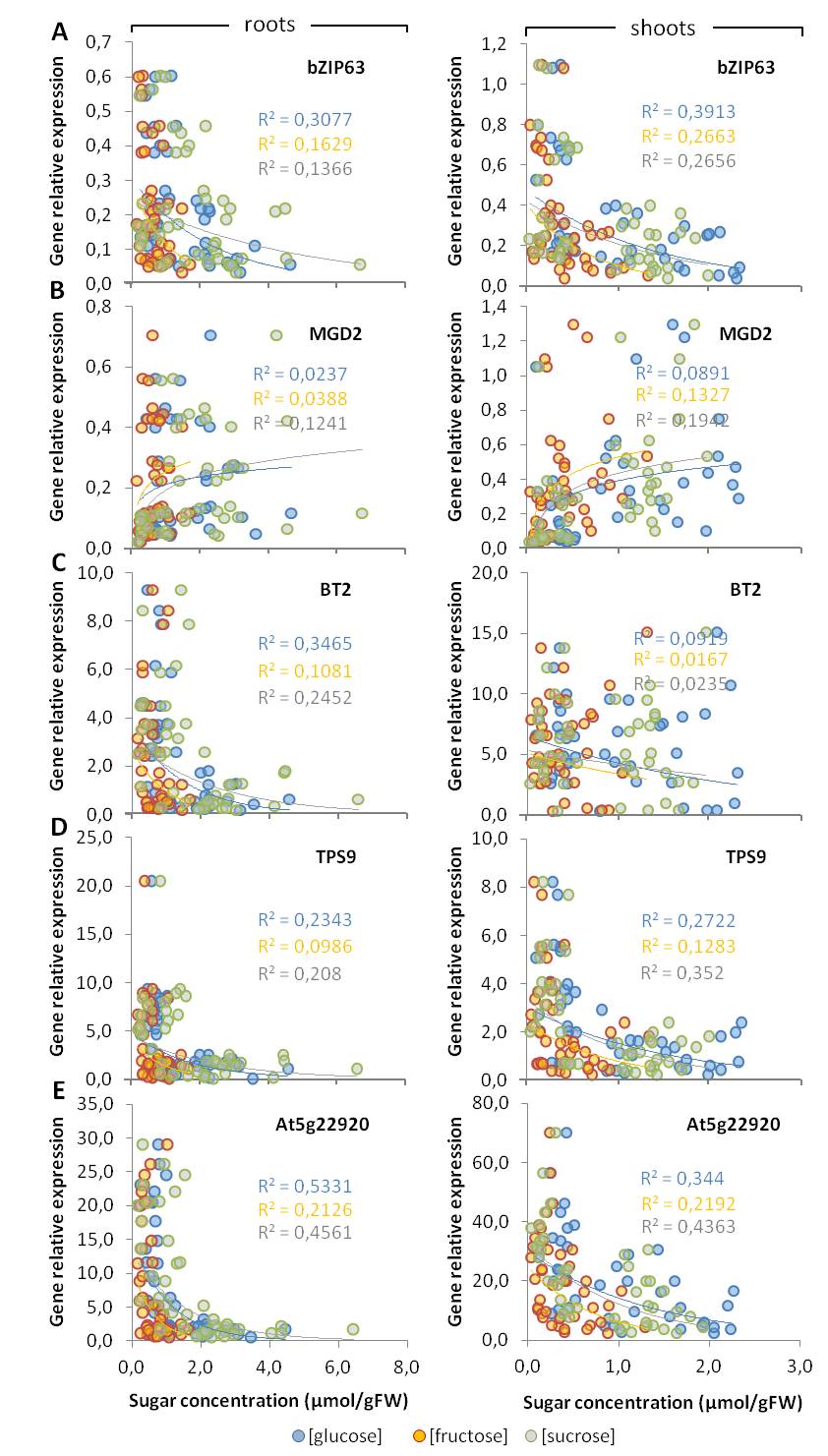

Supplement: Figure S1 — A relationship between internal Glc, Fru, and Suc content and expression of selected genes in solid-medium-grown Arabidopsis plants under steady-state conditions. Standard response curves between sugar content (Glc in blue, Fru in red, and Suc in green) and gene expression were drawn for roots (left column) and shoots (right column) of 13 days old A. thaliana wt and mutants impaired in CH-metabolism at the end of the day and the end of a 6 h extended night. Genes monitored included bZIP63 (A), MGD2 (B), BT2 (C), TPS9 (D), and At5g22920 (E). Correlation coefficients were given for each sample between a specific sugar variation and gene expression. The data were collected from Figures 1, 2. [file Image1.JPEG]

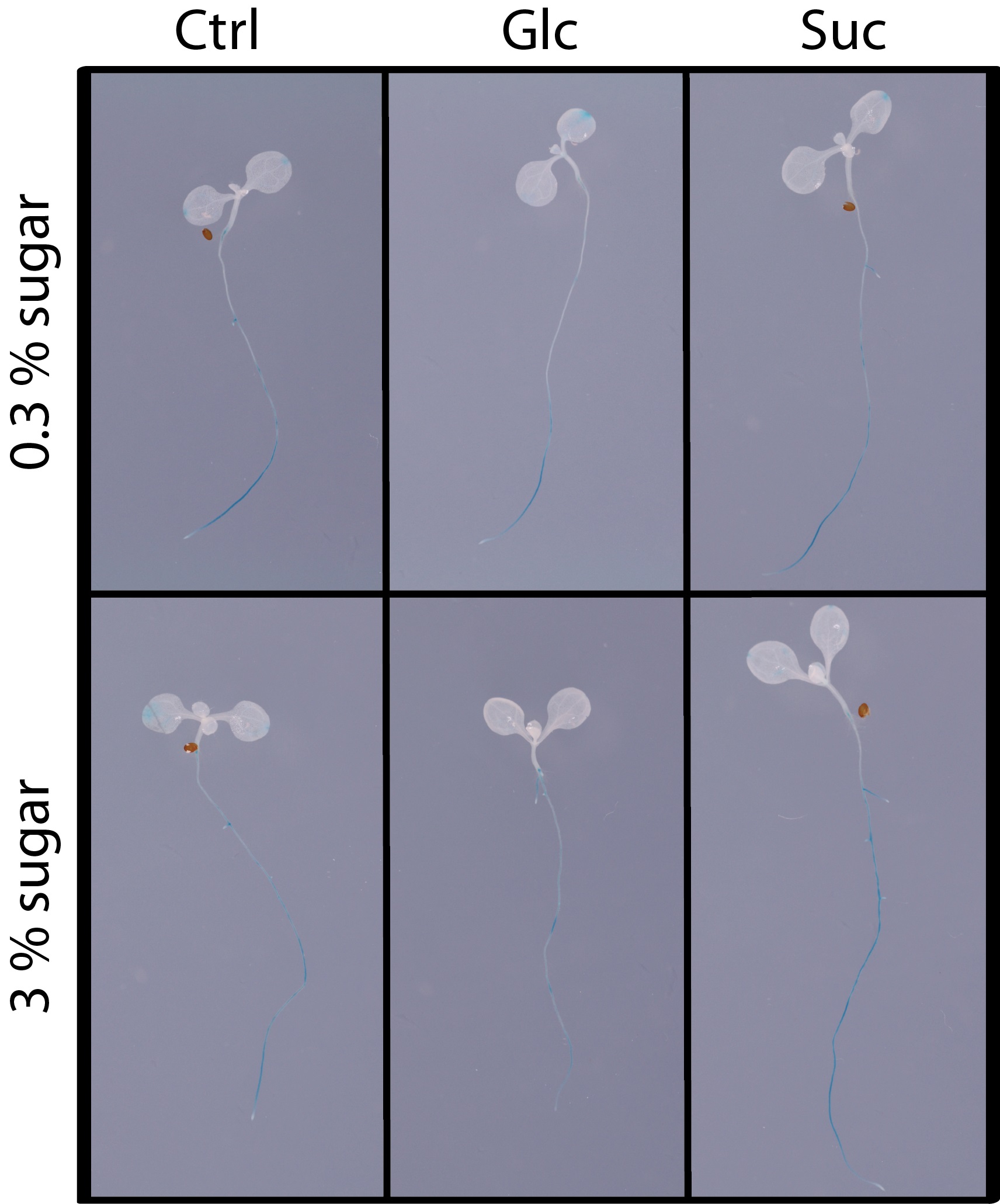

Supplement: Figure S2 — Effects of sugars added to the agar growth medium on expression of bZIP63 in the pbZIP63::GUS line. A. thaliana seedlings were germinated and grown (under a 16 h light/8 h dark photoperiod) for 7 days on solid 0.5× MS agar media containing either no sugar, or Glc (0.3 or 3%) or Suc (0.3 or 3%), and the expression of bZIP63 was visualized by GUS staining. [file Image2.JPEG]

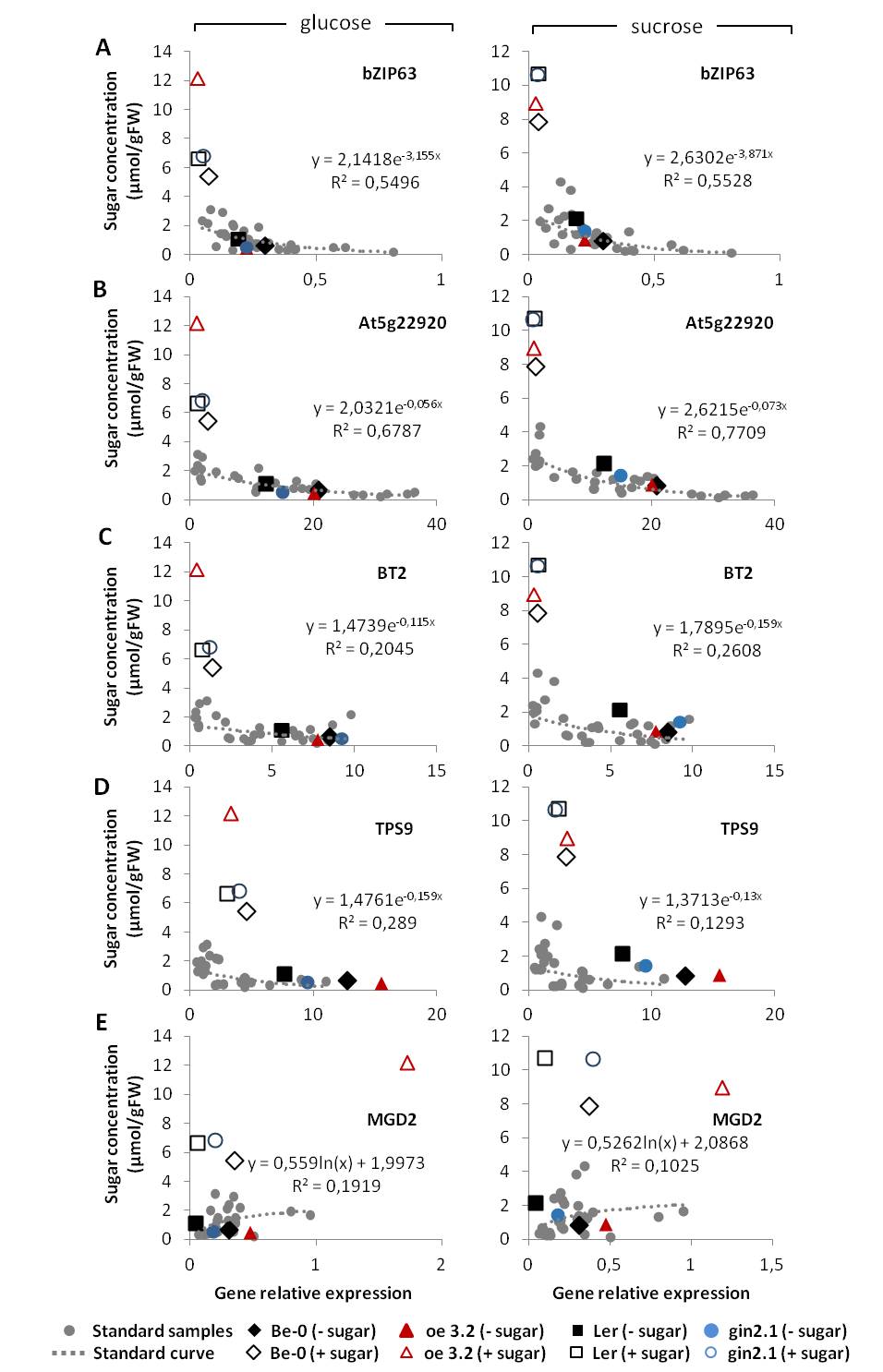

Supplement: Figure S3 — A relationship between internal Glc and Suc content and expression of selected genes in liquid-media-grown Arabidopsis wt plants and HXK1-transgenics (gin2.1 mutant and oe3.2 overexpressor) exposed to external sugar. The plots were generated based on data from Figures 1, 2, 5, 6. Data points indicated by gray circles and spots represent “standard samples” and “standard curves,” respectively, and show the correlation between the averaged sugar and gene expression data from plants grown on solid media under steady state condition. The underlying data are derived from Figures 1, 2. Data points indicated by black, red and blue marks show the relation between sugar content and gene expression in wt and HXK1-transgenics. The underlying data are derived from Figures 5, 6. Please note that, for genes down-regulated by sugars (A–D), the expression was clearly responsive to both Glc and Suc; however, it was only Glc, but not Suc, which at high concentration elicited an enhanced response in oe3.2 overexpressor (when compared to its Be-0 wt control). For MGD2 (E), the expression was less sensitive to sugar content, with the exception of oe3.2 plants where effects of both Glc and Suc were enhanced. Please note also that, at high sugar concentration, the data for TPS9 and MGD2 are considerably shifted from their respective “standard curves.” [file Image3.JPEG]
