# Supplementary material for: Hexokinase 1 is required for glucose-induced repression of bZIP63, At5g22920, and BT2 in Arabidopsis
Source: Front Plant Sci. 2015 Jul 14;6:525. doi: 10.3389/fpls.2015.00525 (PMC4500909; doi:10.3389/fpls.2015.00525)
Supplement: Supplimentary Table 1 — List of qPCR primers. [file Table1.PDF]

Table S1. List of primers used in this study.

AGI number	gene		primer sequence (5' to 3')
AT5G28770.1	bZIP63	forward	TCTTGCTTCTTCCAAAGCTACACC
		reverse	TCACCAGAGAGCTCAGATCCAC
AT5G22920.1	zinc finger protein	forward	GGCGCTATGCATCACAATTGCC
		reverse	AGCATACAGGGCATGTGTATCGG
AT5G20410.1	MGD2	forward	ATGGATGGGAGCTTGTGACTGC
		reverse	GGCACATTCCCTTTCTCCTGTC
AT1G23870	TPS9	forward	ACTCGCAAATGAGCCTGTAGTCG
		reverse	TAGACCTTTGCTTACTCCCTGTGG
AT3g48360	BT2	forward	TCCCTCTCTGCAGGCAATTTAGG
		reverse	CACCAGAAGCTTCCACTTGGTG
AT1G13320	PP2A	forward	TAACGTGGCCAAAATGATGC
		reverse	GTTCTCCACAACCGCTTGGT
AT4G29130	HXK1	forward	GCAGACTTCTCTGTCCTCTGGTAG
		reverse	TCCAACAACATCTTGTCCAACTGC
AT2G19860	HXK2	forward	ACTTCCTCCGCTCAACGCAAAC
		reverse	CTTTGTCGAACGCCACGGTAAC
AT3G27690.1	CAB	forward	ACTCCTCAGAGCATCTGGTACG
		reverse	TTTCTGGATCGGCTGAGAGACC
